# Sperm parameter variability in Donggala bulls: An objective-based analysis of frozen semen quality

**DOI:** 10.5455/javar.2025.l945

**Published:** 2025-08-18

**Authors:** Mardiah Mangun, Yulius Duma, Abdullah Baharun, Ristika Handarini, Syahruddin Said, Widya Pintaka Bayu Putra, Ekayanti Mulyawati Kaiin, Berlin Pandapotan Pardede, Tulus Maulana

**Affiliations:** 1 Faculty of Animal Husbandry and Fisheries, Tadulako University, Central Sulawesi, Indonesia; 2 Department of Animal Science, Faculty of Agriculture, Djuanda University, Bogor, Indonesia; 3 Research Center for Applied Zoology, National Research and Innovation Agency, Bogor, Indonesia

**Keywords:** CASA, donggala bulls, flowcytometry, MMP, sperm quality

## Abstract

**Objective:**

This study aimed to evaluate the properties of fresh and frozen semen from Donggala bulls at the Regional Technical Service Unit of Livestock Breeding in Sidera District, Central Sulawesi.

**Materials and Methods:**

Fresh semen from six selected Donggala bulls was collected using an artificial vagina during the same collection period and weather conditions once per week for four consecutive weeks. Subsequently, the semen was assessed for sperm quality, including volume, pH, mass movement, motility, and concentration. Frozen semen produced from the ejaculation of each bull was used for further analysis (motility, viability, membrane integrity, morphology, and sperm acrosomal status).

**Results:**

Fresh semen parameters, including volume, mass movement, sperm motility, and concentration, differed significantly (*p* < 0.05) among individual Donggala bulls. In contrast, analysis of frozen-thawed semen showed no significant variations among individual bulls regarding sperm motility, abnormality, or membrane integrity, except for sperm viability and acrosomal integrity. Bulls with ID No. 102 and Bull 104 had significantly lower sperm viability (*p* < 0.05) than Bull No. 103. There was also a significant difference (*p* < 0.05) in sperm acrosome integrity between bulls. The results of analysis by computer-assisted sperm analysis (CASA) showed significant differences (*p* < 0.05). In the kinematic parameters of Donggala bull sperm, particularly in velocity straight line, distance straight line, beat cross frequency, and amplitude of lateral head displacement. In contrast, mitochondrial membrane potential (MMP) did not significantly differ (*p* > 0.05) among bulls.

**Conclusion:**

All Donggala bulls showed adequate fertilization ability and good MMP. Flow cytometry and CASA integration provide a rapid and unbiased approach to sperm quality assessment.

## Introduction

Indonesia is known for its rich biodiversity, which includes various livestock species, notably cattle. Among the local cattle breeds, Donggala cattle, recently recognized as an official breed in Central Sulawesi, have garnered attention due to their resilience and reproductive efficiency [1]. These cattle, recognized through the Decree of the Minister of Agriculture of Indonesia, No. 666/Kpts/SR.120/6/2014, share significant physical and genetic similarities with the Ongole crossbreed cattle, such as color, horns, hump, and wattles, likely due to the genetic influence of Ongole cattle [2].

The Donggala cattle exhibit several advantageous traits, including resistance to heat and disease, ease of maintenance, and high reproductive performance. These characteristics make them a valuable breed for local farming practices, especially in tropical environments [1]. To capitalize on these advantages, artificial insemination (AI) programs using frozen semen from superior Donggala bulls have been implemented in Indonesia to enhance livestock populations and genetic quality [3].

Despite the recognized benefits of Donggala cattle and the efforts to improve their population through AI programs, the current methods of assessing sperm quality are predominantly conventional, relying on subjective macroscopic and microscopic evaluations [4]. These methods are prone to inaccuracies, potentially leading to suboptimal selection of bulls for breeding programs. High-quality sperm is crucial for successful fertilization, and as noted by Agnihotri et al. [5], sperm motility, which is vital for fertilization, depends significantly on mitochondrial membrane (MMP) integrity. However, conventional methods do not comprehensively assess these critical parameters.

This knowledge gap demonstrates the need for more objective and accurate methods to evaluate sperm quality, particularly for Donggala cattle, where the limited population further necessitates precise assessments to ensure the success of breeding programs. Recent advancements, such as computer-assisted semen analysis (CASA) [6] and flow cytometry [7], offer more reliable and detailed evaluations of sperm motility and membrane integrity, respectively. These technologies could significantly improve the accuracy of sperm quality assessments, thereby enhancing the effectiveness of AI programs in Donggala cattle.

Therefore, this study aims to comprehensively analyze the sperm quality of Donggala bulls, including fresh and frozen semen, using advanced techniques such as CASA and flow cytometry. The application of these objective-based assessments is expected to provide accurate data on sperm quality, which will serve as a foundation for selecting superior Donggala bulls for AI programs. This research not only addresses the existing gaps in sperm quality evaluation but also contributes to the overall improvement of cattle breeding practices in Indonesia.

## Materials and Methods

### Ethical approval

The Animal Ethics Commission of the National Research and Innovation Agency (BRIN) approved this study's animal models and experimental designs (certificate No.: 049/KE.02/SK/03/2023).

### Study design and experimental animals

All Donggala bulls used were aged 5 to 7 years at the Regional Technical Service Unit of Livestock Breeding in Sidera District, Central Sulawesi. This breeding center is the only institute developing Donggala bulls in Indonesia. Due to the limited semen quality assessment facilities and tools, only fresh semen quality testing was conducted. In contrast, frozen semen quality assessment was performed at the Genomic Laboratory, National Research and Innovation Agency (BRIN). Through the application of an artificial vagina, fresh semen was successfully retrieved from a total of six Donggala bulls during the same collection period and weather conditions once per week for four consecutive weeks. Subsequently, the semen was assessed for sperm quality, including volume, pH, mass movement, motility, and concentration. This was followed by cryopreservation using the same extender, AndroMed^®^. Frozen semen produced from the ejaculation of each bull was used for further analysis.

### Fresh semen quality assessment

Parameters of fresh semen quality assessed at this stage included volume, pH, mass movement, motility, and concentration. The measurement of fresh semen volume involved measuring the scale value on the collection tube. The pH assessment was conducted by observing the color change on special pH indicator paper (Merck, Mquant^®^) dripped with semen, at a pH scale of 6.4 to 8.0. To assess sperm mass movement, semen was dripped on a slide and observed under a microscope (BOECO Binocular Microscope, BM-700) with 100x magnification. This assessment was carried out by examining the thickness of the mass wave and the speed of its movement. The criteria for assessing sperm mass movement included (0): no sperm movement; (+): weak sperm mass movement and few waves; (++): fast sperm mass movement and thick waves; (+++): swift movement of the sperm mass and thick waves.

Assessment of sperm motility in fresh semen involved dispensing fresh semen in small droplets onto a slide with the addition of the saline solution, which was subsequently overlaid with a coverslip and examined using a microscope (BOECO Binocular Microscope, BM–700) at a magnification of 400x. Microscopic analysis was performed, and the sperm concentration was calculated employing the Photometer SDM 6 device (Minitube, Germany) with a wavelength of 546 nm and referred to standard procedures. Subsequently, a total of 35 μl of fresh semen and 3.5 ml of 0.9% NaCl were mixed slowly, transferred to a cuvette, and put into a photometer to calculate the concentration in units of ×10^9^ sperm cells per ml.

### Cryopreservation and frozen semen quality assessment

The semen was diluted using a Tris-egg yolk extender composed of Tris aminomethane (3.634 gm), D-glucose (0.50 gm), 20% egg yolk, glycerol, and penicillin-streptomycin (penstrep) antibiotics to achieve a final concentration of 25 million spermatozoa per 0.25 ml mini straw. After dilution, the semen underwent an equilibration phase at 4°C lasting between 2 and 4 h. After being arranged on a dynamic rack, the straws were subjected to nitrogen vapor freezing 5 cm above liquid nitrogen for 15 min, then submerged into –196°C liquid nitrogen for long-term preservation.

After immersing the straws in a 37°C water bath for 30 sec to thaw, both ends were cut open and the semen was transferred into a microtube. Throughout the analysis, the thawed semen was held at a constant 37°C. Evaluations were performed to assess sperm viability, morphology, plasma membrane functionality, and acrosomal integrity.

A 10 μl semen sample was the starting point for evaluating sperm viability and morphology, utilizing the eosin-nigrosin staining technique as the diagnostic medium on a glass slide and mixed with a staining solution containing 0.2 gm eosin and 2 gm nigrosin in 100 ml of distilled water. The mixture was homogenized, smeared onto the slide, and dried on a warm plate. Microscopic examination under 400x magnification differentiated viable (unstained) and non-viable (stained) spermatozoa. Viability was determined by counting 200 sperm cells. Morphological evaluation involved observing 200 spermatozoa and distinguishing between normal and abnormal forms [8].

Using the HOS assay, the functional integrity of the sperm plasma membrane was evaluated. A volume of 30 μl of semen was added to 300 μl of hypo-osmotic solution formulated with 0.735 gm sodium citrate and 1.351 gm fructose per 100 ml of distilled water. The sample was incubated in a 37°C water bath for 30 min. Subsequently, a single drop was mounted on a slide, sealed with a coverslip, and analyzed microscopically at 400x. Spermatozoa with preserved membranes exhibited characteristic tail coiling. Out of 200 spermatozoa, reactive and non-reactive cells were enumerated [9].

**Figure 1.**
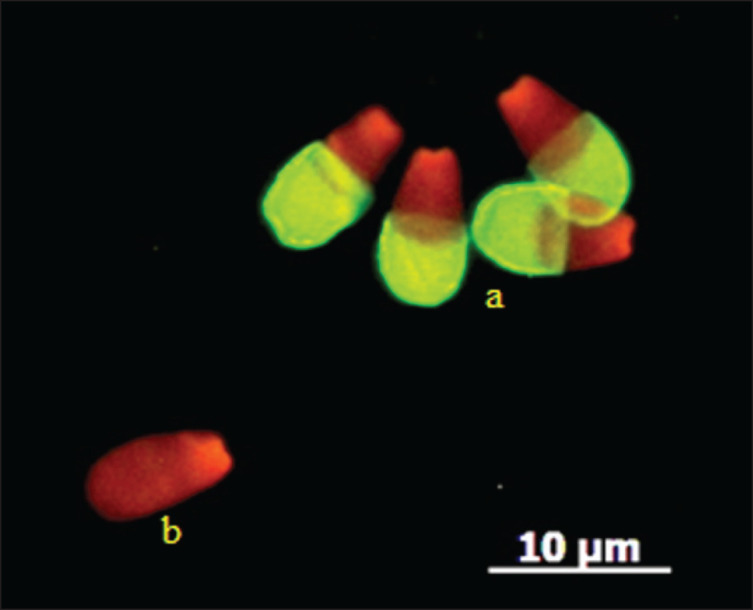
Magnification 630x, microscope fluorescence, Imager Z2, Carl Zeiss, Germany. Acrosomal status: (a) Intact acrosome (green fluorescence in the acrosome), (b) Non-intact acrosome (red fluorescence, no acrosome).

The acrosome status was determined using a fluorescent labeling method involving FITC–PNA Sigma and PI. Semen samples were air-dried at room temperature and fixed with 96% ethanol for 10 min. A volume of 30 μl FITC-PNA (100 μg/ml). After the addition, the mixture was incubated at 37°C for 30 min. Followed by the application of 5 μl PI (1 μg/μl), which was applied and incubated for 5 min. Slides were rinsed three times with phosphate-buffered saline and then covered with coverslips. The evaluation was performed using a fluorescence microscope at 380–420 nm. A total of 200 spermatozoa were counted per treatment. Green fluorescence signified intact acrosomes, while red fluorescence indicated damaged ones (Fig. 1). All evaluations were conducted in a dark room [9].

### Objective-based frozen semen quality tests

In this study, objective-based frozen semen quality tests were conducted to assess sperm motility, movement patterns (kinematics), and MMP. Using the CASA method, sperm motility and movement patterns were evaluated with the aid of SpermVision^TM^ 3.7.8 software (Minitube, Germany). The system was connected to a Carl Zeiss Microimaging GmbH unit (Gottingen, Germany) with a heated stage maintained at 38°C. A 5 μl semen sample was prepared on a glass slide and covered for observation under the microscope. Subsequently, between 750 and 1,000 sperm cells were assessed across eight microscopic fields, following the default factory settings for bull sperm analysis. Various sperm motion parameters were measured using the CASA system, including distance average path (DAP), distance curve line (DCL), and distance straight line (DSL). It also recorded motility indicators such as progressive motility, velocity of the average path (VAP), velocity straight line (VSL), and curvilinear velocity (VCL). Other metrics included beat cross frequency (BCF), wobble (WOBB), amplitude of lateral head displacement (ALH), straightness (STR), and mean linearity (LIN) [10].

**Figure 2.**
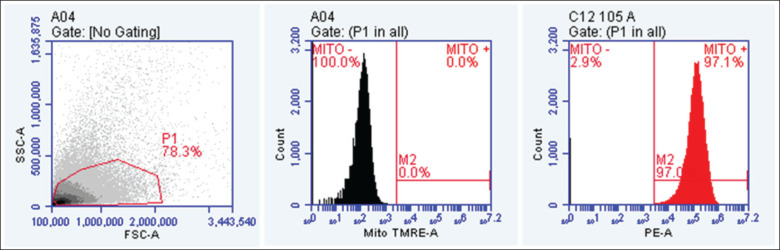
MMP analysis using BD Accuri 6 Plus flow cytometer (Becton Dickinson, San Jose, CA). A- The study collected data on the intensity of forward-scattered (FSC) and side-scattered (SSC) light. To eliminate signals originating from cellular debris or cells displaying abnormal morphology, threshold levels for the side-scatter area (SSC-A) and forward-scatter area (FSC-A) were established. B- Unstained cells as control. (c) Cells with mito TMRE staining appeared with high peaks on different sides.

The sperm MMP was evaluated using the BD Accuri 6 plus flow cytometer (Becton Dickinson, San Jose, CA), with the TMRE MitoStatus staining kit (Tetramethylrhodamine ethyl ester, BD Pharmingen, USA). Semen with a maximum sperm concentration of 1×10^6^ cells/ml was mixed in pre-warmed cell culture medium, stained with 200 nM MitoStatus TMRE, and incubated for 30 min at 37°C. The stained sperm sample was washed twice with BD Pharmingen^TM^ Stain Buffer (FBS), and the supernatant was decanted and mixed gently to disrupt the sperm pellet. Subsequently, the sample was resuspended in the Stain Buffer (FBS) and analyzed using a flow cytometer (575/26 or 582/15 nm, BD Accuri^TM^ C6 Plus, B.D., USA) at a low flow rate of 10–20 µl/min [11]. The acquisition was terminated after 50,000 events were recorded, and data collected during the acquisition were recorded in list mode to enable detailed subsequent analysis, as presented in Figure 2.

### Statistical analysis

Data processing in this research were carried out using Minitab Statistical Software version 18.1 (Minitab, USA). The assumption of normality was examined through the Shapiro–Wilk test, while Levene’s test assessed the equality of variances. As the dataset exhibited normal distribution but lacked homogeneity, an ANOVA was implemented. Significant intergroup variations were evaluated through Tukey’s post hoc test, with all results reported as mean ± standard deviation.

## Results

A total of six bulls from the Donggala breeding center were used in this study, each with corresponding codes 102, 103, 104, 105, 110, and 111. As presented in Table 1, the results of the fresh semen quality assessment varied between individuals, particularly in volume (*p < *0.05), motility (*p < *0.05), and sperm concentration (*p < *0.05). Furthermore, the sperm pH values of all tested bulls were the same, and the mass movement of bulls 102 and 103 was significantly different (*p < *0.05) compared to the others. The lowest fresh semen quality values of the several parameters tested were shown in the bulls' ID Nos. 105, 111, and 102, as shown in Table 1.

The percentage of sperm viability and acrosome status varied significantly (*p < *0.05) between individual Donggala bulls. As shown in Table 2, bull 104 exhibited the lowest viability percentage, and the highest acrosome status was for bulls 105 and 110. The assessment of kinematic patterns of sperm results in each Donggala bull using objective-based frozen semen quality tests, CASA, was presented in Table 3. The results of evaluating the kinematic patterns of sperm in each bull did not show a significant difference (*p *> 0.05) in the VAP, VCL, DAP, DCL, LIN, STR, and WOBB. Meanwhile, VSL, DSL, BCF, and ALH values were different (*p < *0.05) among Donggala bulls.

The results of the MMP assessment of sperm in each Donggala bull using objective-based frozen semen quality tests based on a flow cytometer are shown in Table 4. There was no significant difference (*p *> 0.05) in the percentage of MMP between Donggala bulls.

## Discussion

In this study, the motility of frozen sperm has met the minimum standard of 40% for post-thaw frozen semen [12]. pH of semen from Donggala bulls was found to be 6.5, which falls within the normal range of 6.4–7.8 as reported by Garner and Hafez [13]. Maintaining a balanced pH is vital for preserving the stability of the seminal plasma environment, which supports proper sperm function. Disruptions such as bacterial contamination or the accumulation of lactic acid, a metabolic byproduct of fructolysis, can shift the pH toward acidity, thereby compromising sperm motility and overall functionality [14, 15]. Therefore, it is crucial to ensure a stable pH in semen for preserving fertility, especially in the context of AI practices.

The sperm concentration in this study varied significantly among the Donggala bulls, with Bulls 111 and 110 showing notably higher concentrations than Bulls 102 and 105. Contrary to the negative correlation between semen volume and sperm concentration reported by Mapel et al. [16], the results indicate a positive correlation, implying that elevated sperm concentrations may reflect a greater reproductive potential. Nonetheless, such variations could be attributed to factors like genetic background, testicular volume, and the frequency of semen collection. For instance, bulls with larger testes and more frequent collections may produce ejaculates with higher sperm counts. Despite this, it is crucial to recognize that increased sperm concentration does not always equate to better fertility outcomes, as it may also contribute to elevated oxidative stress. This phenomenon was exemplified by Bull 111, which, despite its high sperm count, exhibited reduced motility and compromised acrosomal integrity.

**Table 1.** Fresh semen quality of Donggala bull.

Bulls ID	Sperm parameters (Mean ± SEM)				
	Semen volume (ml)	pH	Mass movement (Score 0–3)	Motility (%)	Concentration (×10^6^)
102	5.33 ± 0.57^ab^	6.5	2.66 ± 0.57^a^	80 ± 0^a^	1319.33 ± 112.0^c^
103	6.93 ± 1.0^ab^	6.5	2.33 ± 0.57^a^	76.66 ± 5.77^a^	1516.66 ± 63.50^ab^
104	5.16 ± 1.25^ab^	6.5	2 ± 0^b^	81.66 ± 2.88^a^	1553.33 ± 130.12^ab^
105	4.66 ± 0.57^b^	6.5	2 ± 0^b^	80 ± 0^a^	1370 ± 131.14^bc^
110	6.23 ± 1.07^ab^	6.5	2 ± 0^b^	70 ± 0^b^	1601 ± 108.44^a^
111	7.5 ± 2.59^a^	6.5	2 ± 0^b^	66.66 ± 5.77^b^	1644 ± 38.57^a^

Note: ^a,b,c^Means in the same column with different superscripts differ significantly (*p < *0.05).

**Table 2.** Frozen semen quality of the Donggala bull.

Bulls ID	Sperm parameters (Mean% % ± SEM)				
	Motility	Viability	Abnormality	I.M	Acrosomal status
102	53.6 ± 5.6	47.6 ± 9.1^b^	8.3 ± 1.7	50.3 ± 6.1	91.6 ± 0.9^b^
103	55.8 ± 8.4	59.4 ± 7.4^a^	5.6 ± 2.4	51.0 ± 8.1	96.4 ± 3.3^a^
104	47.5 ± 4.6	46.6 ± 5.8^b^	8.6 ± 2.6	43.4 ± 4.3	94.4 ± 2.7^ab^
105	53.6 ± 9.3	53.6 ± 9.3^ab^	5.9 ± 1.7	47.5 ± 5.2	97.4 ± 0.8^c^
110	47.7 ± 5.6	54.1 ± 6.4^ab^	7.9 ± 1.7	44.5 ± 4.5	97.4 ± 0.8^c^
111	51.2 ± 3.2	52.6 ± 4.1^ab^	6.9 ± 3.1	48.0 ± 4.6	95.8 ± 2.7^a^

Note: ^a,b,c^Means in the same column with different superscripts differ significantly (*p < *0.05). I.M = Membrane integrity.

**Table 3.** The results of sperm kinematic pattern assessment in each Donggala bull using CASA frozen semen.

Parameters	Bulls (Mean ± SEM)					
	102	103	104	105	110	111
VAP (µm/sec)	69.6 ± 9.3	61.9 ± 5.9	68.5 ± 5.8	66.3 ± 6.8	67.0 ± 11.2	67.0 ± 4.4
VCL (µm/sec)	94.0 ± 16.1	82.5 ± 9.6	92.1 ± 9.2	91.7 ± 11.9	92.4 ± 18.1	90.3 ± 4.1
VSL (µm/sec)	46.9 ± 6.4^ab^	39.8 ± 0.9^b^	45.0 ± 3.4^ab^	43.7 ± 2.3^ab^	47.6 ± 9.2^a^	44.9 ± 2.4^ab^
DAP (µm/sec)	28.8 ± 4.0	24.9 ± 2.8	27.9 ± 2.3	27.6 ± 2.8	28.2 ± 5.1	27.9 ± 1.3
DCL (µm/sec)	39.0 ± 6.8	33.2 ± 4.4	37.6 ± 3.7	38.3 ± 4.8	39.0 ± 8.1	37.6 ± 0.7
DSL (µm/sec)	19.6 ± 3.0^a^	15.7 ± 0.6^b^	18.2 ± 1.5^ab^	18.1 ± 1.0^ab^	20.0 ± 4.2^a^	18.8 ± 0.8^ab^
LIN (%)	53.0 ± 8.9	48.0 ± 5.4	49.0 ± 2.2	47.8 ± 4.3	51.0 ± 2.2	57.5 ± 11.7
STR (%)	68.0 ± 7.5	64.3 ± 5.3	65.5 ± 1.7	65.8 ± 3.9	70.3 ± 2.9	67.3 ± 1.0
WOBB (%)	74.3 ± 2.6	74.5 ± 1.7	74.3 ± 1.7	72.0 ± 2.9	72.3 ± 2.1	74.0 ± 0.8
BCF (Hz)	22.6 ± 3.7^ab^	19.1 ± 2.1^b^	22.2 ± 2.8^ab^	23.2 ± 1.8^a^	24.8 ± 3.5^a^	23.7 ± 1.6^a^
ALH (µm)	6.0 ± 0.7^a^	5.6 ± 0.1^ab^	5.6 ± 0.3^ab^	5.0 ± 0.7^bc^	4.8 ± 0.5^c^	5.7 ± 0.4^ab^

Note: ^a,b^Means in the same row with different superscript differ significantly (*p < *0.05). ALH = Amplitude of lateral head displacement; BCF = beat cross frequency; DAP = Distance average path; DCL = Distance curve length = DSL = Distance straight length; LIN = Linearity; VAP = Velocity average path; VCL = Velocity curve length; VSL = Velocity straight length; STR = Straightness; WOBB = Wobble.

Although Bull 111 had a high sperm concentration (1644 ± 38.57 × 10^6^ cells/ml), its sperm motility (66.66% ± 5.77%) and acrosomal integrity (95.8% ± 2.7%) were comparatively lower than those of other bulls. Sperm concentration in bull ejaculates is generally influenced by factors such as testicular size and the frequency of semen collection. Research on Bali bulls, for instance, has demonstrated that scrotal circumference is favorably correlated with ejaculate volume, sperm concentration, and progressive motility [17]. The volume, color, consistency, and concentration of sperm are interconnected factors. This means that semen coloration is influenced by sperm concentration levels, which is also reflected in the consistency of the sperm [9].

**Table 4.** Mitochondrial membrane potential (MMP) of sperm in Donggala bull (Mean ± SEM).

Bulls ID	MMP (%) (Mean ± SEM)
102	99.40 ± 0.14
103	92.75 ± 8.98
104	98.90 ± 0.14
105	98.15 ± 1.48
110	98.55 ± 0.07
111	99.15 ± 0.21

The inverse association between sperm count and associated quality metrics, with motility and acrosomal integrity, is often linked to oxidative stress. Elevated sperm concentrations tend to coincide with increased oxidative activity, which may contribute to greater incidences of sperm abnormalities and a reduction in both motility and acrosomal function [18]. ROS, cellular oxidative metabolism, produces reactive byproducts that can induce substantial genotoxic effects on sperm DNA and are implicated in a range of morphological deformities that adversely affect fertility [19].

Acrosomal integrity plays a pivotal role in determining the fertilizing capacity of sperm. It is directly involved in the AR, a critical event required for successful oocyte penetration. In the present study, semen from Donggala bulls demonstrated acrosomal integrity within the acceptable range, meeting the minimum threshold of 65% as outlined by the Indian Ministry of Agriculture.

The process known as the acrosome reaction entails the convergence of the acrosomal membrane with the overlying plasma membrane, a crucial step that facilitates the enzymatic breakdown of the zona pellucida through the release of hydrolytic enzymes. This process is tightly regulated by intracellular signals such as calcium ion concentration (Ca^2+^), intracellular pH shifts, and changes in membrane potential [20]. Monitoring acrosomal integrity is thus essential, as premature or incomplete acrosome reactions can compromise the sperm's fertilization potential. For this reason, assessing this parameter should remain a core component of comprehensive semen evaluation protocols.

The acrosome reaction represents a vital exocytotic process enabling the enzymatic release necessary for sperm to traverse the zona pellucida and achieve fertilization. The structural integrity of the acrosome is essential, as premature or incomplete reactions can compromise sperm functionality and result in infertility. Simons and Fauci [21] emphasized the complexity of this process, noting its reliance on calcium ion influx and the precise orchestration of membrane fusion events. Traditionally, this reaction is believed to be initiated upon the sperm’s contact with the zona pellucida.

In this study, the acrosomal status was evaluated using the PNA assay, a widely accepted method for assessing acrosomal integrity and, by extension, semen quality. Evaluating the integrity of the acrosome is particularly crucial in the context of male infertility, as spermatozoa lacking functional acrosomes are incapable of fertilizing the oocyte. Accurate semen analysis, therefore, must incorporate acrosomal assessment to ensure reliable fertility diagnostics [22].

Sperm motility also plays a decisive role in reproductive success, influencing the sperm’s capacity to reach and fertilize the oocyte. Significant differences in sperm kinematic parameters were observed among Donggala bulls, particularly in metrics such as VSL, VCL, BCF, and ALH [23]. These parameters are key indicators of sperm fertilization potential, with specific threshold values—such as VCL > 70 µm/sec and both VAP and VSL > 45 µm/sec—serving as benchmarks for effective sperm motion [24]. Sperm exhibiting BCF values above 20 Hz and ALH values within the 2.5–6.5 µm range are considered to possess optimal motility characteristics [25]. In this study, Bull 103 demonstrated lower VSL and BCF values relative to its peers, suggesting a reduced likelihood of successful fertilization. These findings underscore the necessity of detailed motility profiling within semen evaluation protocols.

Beyond motility, mitochondrial functionality significantly influences sperm quality. The MMP serves as a reliable biomarker of both sperm viability and fertilization capacity. Prior research has demonstrated that Nellore bulls with elevated MMP levels achieved higher conception rates (65%) compared to those exhibiting diminished mitochondrial activity (36%) [26]. This suggests that mitochondrial performance, through its effect on motility, is intricately linked to male fertility. In the present study, the dual assessment of DNA fragmentation and MMP in infertile males emerged as a promising approach to predict the likelihood of natural conception, even when standard semen parameters appear normal.

Evaluating mitochondrial function thus offers crucial insights into the energy dynamics and functional competence of sperm cells. Furthermore, this parameter can be employed to assess the efficacy of various semen extenders, thereby contributing to better preservation strategies and improved reproductive outcomes.

Despite its valuable contributions, this study is not without limitations. One major constraint is the ability to extrapolate these results to the overall Donggala bull population, which remains constrained. Moreover, while the study provided an in-depth analysis of key sperm parameters such as motility, concentration, and mitochondrial health, it did not incorporate other influential factors like genetic variability or hormonal profiles, which could also play significant roles in determining male fertility.

## Conclusion

In conclusion, this study showed that the fresh and frozen semen characteristics exhibited variation between Donggala bulls. However, all Donggala bulls exhibited the ability to fertilize and had favorable MMP. The integration of flow cytometry and CASA offered an accurate and objective method for analyzing sperm quality. This combination enabled efficient assessment and evaluation of various parameters related to sperm quality, enhancing the understanding and improvement of reproductive performance in Donggala bulls.

## List of Abbreviations

ALH, amplitude of lateral head displacement; AO, acridine orange; ATP, adenosine triphosphate; AV, artificial vagina; BCF, beat cross frequency; DAP, distance average path; DCL, distance curve line; DSL, distance straight line; FITC–PNA, fluorescein isothiocyanate–peanut agglutinin; HOST, hypo-osmotic swelling test; LIN, mean linearity; PM, progressive motility; PRM, protamine; ROS, reactive oxidative stress; STR, straightness; TM, total motility; VAP, velocity of the average path; VCL, curvilinear velocity; VSL, velocity straight line; WOBB, wobble; ZP, zona pellucida.
